# A Mobile App and Dashboard for Early Detection of Infectious Disease Outbreaks: Development Study

**DOI:** 10.2196/14837

**Published:** 2021-03-09

**Authors:** Euijoon Ahn, Na Liu, Tej Parekh, Ronak Patel, Tanya Baldacchino, Tracy Mullavey, Amanda Robinson, Jinman Kim

**Affiliations:** 1 School of Computer Science The University of Sydney Darlington Australia; 2 Telehealth Technology Centre Nepean Hospital Nepean Blue Mountains Local Health District Kingswood Australia; 3 The University Sydney Business School Darlington Australia; 4 Tej Consultancy Sydney Australia; 5 Public Health Unit Nepean Hospital Nepean Blue Mountains Local Health District Kingswood Australia

**Keywords:** public health, infectious disease reporting, mobile app, disease notification, mobile phone

## Abstract

**Background:**

Outbreaks of infectious diseases pose great risks, including hospitalization and death, to public health. Therefore, improving the management of outbreaks is important for preventing widespread infection and mitigating associated risks. Mobile health technology provides new capabilities that can help better capture, monitor, and manage infectious diseases, including the ability to quickly identify potential outbreaks.

**Objective:**

This study aims to develop a new infectious disease surveillance (IDS) system comprising a mobile app for accurate data capturing and dashboard for better health care planning and decision making.

**Methods:**

We developed the IDS system using a 2-pronged approach: a literature review on available and similar disease surveillance systems to understand the fundamental requirements and face-to-face interviews to collect specific user requirements from the local public health unit team at the Nepean Hospital, Nepean Blue Mountains Local Health District, New South Wales, Australia.

**Results:**

We identified 3 fundamental requirements when designing an electronic IDS system, which are the ability to capture and report outbreak data accurately, completely, and in a timely fashion. We then developed our IDS system based on the workflow, scope, and specific requirements of the public health unit team. We also produced detailed design and requirement guidelines. In our system, the outbreak data are captured and sent from anywhere using a mobile device or a desktop PC (web interface). The data are processed using a client-server architecture and, therefore, can be analyzed in real time. Our dashboard is designed to provide a daily, weekly, monthly, and historical summary of outbreak information, which can be potentially used to develop a future intervention plan. Specific information about certain outbreaks can also be visualized interactively to understand the unique characteristics of emerging infectious diseases.

**Conclusions:**

We demonstrated the design and development of our IDS system. We suggest that the use of a mobile app and dashboard will simplify the overall data collection, reporting, and analysis processes, thereby improving the public health responses and providing accurate registration of outbreak information. Accurate data reporting and collection are a major step forward in creating a better intervention plan for future outbreaks of infectious diseases.

## Introduction

Outbreaks of infectious diseases, such as gastroenteritis, measles, influenza, and COVID-19, pose great risks, including hospitalization and death, to public health. Outbreaks have been directly associated with increased utilization of health care resources and negative social impacts [1,2]. Therefore, improving the management of these outbreaks is important for preventing widespread infection and mitigating associated risks. A core strategy to minimize the risk posed by widespread infectious diseases is the development of a surveillance system to quickly identify potential outbreaks as early as possible accurately, such that an effective intervention plan can be implemented before the substantial costs associated with the spread of diseases have been incurred.

Many infectious disease surveillance (IDS) systems have been developed to quickly collect, interpret, and respond to outbreaks. Organizations such as the US Centers for Disease Control and Prevention [3], Germany’s SurvNet@RKI [4], and Australia’s Health Victoria [5] have established digital and web-based disease notification systems. These systems are beneficial for timely reporting of disease outbreaks between distributed sites (often dispersed geographical locations) such as hospitals or community facilities (eg, aged care and childcare centers) and local public health units (PHUs). However, each system has been developed to satisfy particular needs (eg, specific to certain diseases [6] or patients only) and is thus limited in its applicability to a wider domain. Meanwhile, local health authorities in many countries still rely on conventional manual paper forms and communicate them via fax or email (eg, New South Wales [NSW] Ministry of Health [7]). Reporting in such a way between geographically dispersed sites is susceptible to lost, incomplete, and incorrect information [8]. This is problematic because of the increased public health costs associated with infectious diseases.

Mobile health (mHealth) technology is an emerging concept that uses mobile devices coupled with wireless technology for health care purposes [9-11]. It has gained great popularity in recent years and has been used in various clinical environments. Communication between clinicians and hospitals has been improved greatly using *push alert* and automatic notification (eg, paging) features [12]. In addition, a number of innovative mobile apps have been developed, such as remote patient monitoring systems [13] and orthopedic decision support apps [14]. With continued innovation in mHealth technologies, dashboards have also been introduced to summarize and integrate key information into a visual display for better decision making [15-17]. Clinical dashboards are generally designed to improve the quality of care by measuring performance metrics such as the doctor-to-bed ratio, hospital infection rates, and mortality ratios [15]. These data are then shared among clinicians in real time to react to patient needs as well as to efficiently capture and understand underlying patterns and trends.

**Figure 1:**
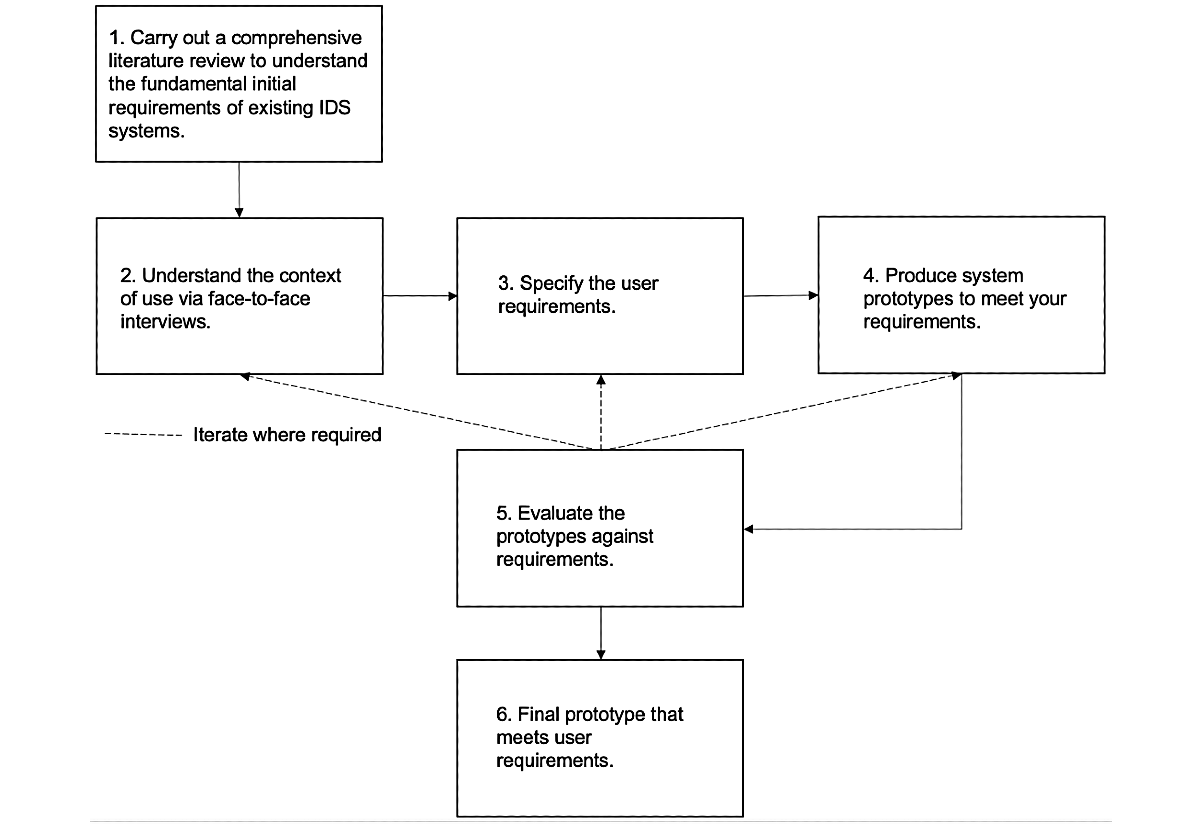
Flowchart of our 2-pronged approach. IDS: infectious disease surveillance.

Limited research has been conducted to investigate the use of mHealth technology coupled with dashboards to improve the management of the outbreak of infectious diseases. In this paper, we propose a new IDS system that accurately captures outbreak data using a mobile app and analyzes them by visualizing interactively using a dashboard. We developed our system using a 2-pronged approach; we firstly carried out a comprehensive literature review to understand the initial fundamental requirements of existing IDS systems and then conducted iterative face-to-face interviews to understand the context of use and collect user requirements. A flowchart of our 2-pronged approach is shown in Figure 1. We suggest that the use of mHealth technology and dashboard can improve the management of outbreaks between distributed sites and identify new opportunities for better health outcomes and planning.

## Methods

### Study Site and Setting

This study was conducted at Nepean Blue Mountains Local Health District (NBMLHD), a Nepean hospital in NSW, Australia. NBMLHD covers both urban and semirural areas, covering approximately 9179 km^2^. The estimated resident population of NBMLHD in 2017 was 381,704 and is projected to increase by 30% by 2036. The PHU at NBMLHD identifies and prevents or minimizes public health risks to the communities by working with local general practitioners, community health workers, schools and childcare centers, and aged care facilities.

All health facilities (eg, childcare centers and aged care facilities) in NBMLHD (and also across the NSW state) currently use a paper-based form to report outbreaks to local PHU teams, which is error prone and time consuming. The increasing populations along with large covering areas of the district will introduce new and unique challenges in the management of infectious disease outbreaks.

### Identification of Fundamental Requirements of the IDS System Through a Literature Review

We conducted a literature review on existing IDS systems to analyze and define the overall scopes and fundamental requirements of our system, following the PRISMA-P (Preferred Reporting Items for Systematic Reviews and Meta-analyses Protocols) guidelines [18]. We also analyzed the design trends and incorporated the relevant technologies into our system, with a focus on studies that used mobile devices and dashboard systems.

**Figure 2:**
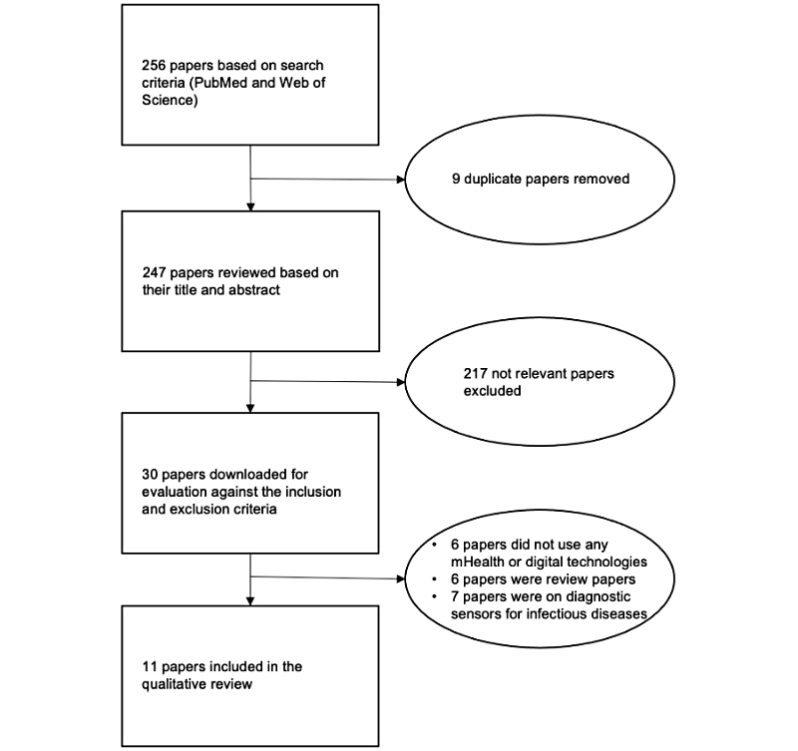
Flowchart of paper selections. mHealth: mobile health.

We conducted a systematic search of published papers from the following scientific databases: PubMed and Web of Science. We searched the papers published in the last 15 years from 2004, concerning infectious disease reporting and analysis using mHealth technologies. The search terms used were “infectious” AND “disease” AND “surveillance” AND “smartphone” OR “smartphone application” OR “mobile app” OR “mobile phones” OR “dashboard” OR “mHealth.” Figure 2 provides an overview of the study selection process.

The inclusion criteria were as follows: (1) infectious disease reporting systems must have been designed to use an electronic device (mobile phone, tablet, or personal computer) and (2) reporting was in the form of a specific app or website. We excluded papers that described the use of mobile-based systems for noninfectious diseases. Review papers were excluded. Only papers published in English were included.

### Identification of Specific User Requirements of the IDS System Through Interviews

Face-to-face interviews were conducted to collect the user requirements of our IDS system. Multiple interviews were carried out iteratively until we had a comprehensive understanding of user requirements. The participants included public health nurses from the local PHU team and telehealth providers from the NBMLHD. The interviews were conducted by experienced researchers, including project coordinators and software developers. First, we identified different types of end users and their corresponding roles. We then investigated and defined specific workflows and use case scenarios of our system. Our study did not require any patient involvement, as ethical approval or informed consent was not needed.

### User Interface Design and Agile Development

Agile development uses an interactive system framework that aims to provide a good user experience. The initial user interfaces of the system were designed based on the user requirements and the understanding of the context of use. The software developers and health care team were in constant communication, discussing and enhancing the contents and user interfaces of the system. We developed a functional mobile app and dashboard prototype that was used to collect further feedback from users.

## Results

### Phase 1: Literature Review of the State of the Art

Our initial search identified 256 papers using the search criteria. We removed 9 duplicate papers and screened the remaining 247 papers based on their titles and abstracts. We then downloaded 30 relevant papers and examined them based on the inclusion and exclusion criteria. A total of 11 relevant papers were selected for qualitative review.

The papers described new digital technologies for better detection of, reporting on, and response to the outbreak of the diseases. A summary of our literature review of the existing IDS systems is shown in Table 1. Through analyzing the common and central aims of selected studies, we have identified 3 fundamental requirements for infectious disease reporting systems, which is the ability to capture and report outbreak data accurately, completely, and in a timely fashion [19-25]. Fundamental requirements were also used as the basis for identifying subsequent specific user requirements.

The ability to accurately capture and report data, such as symptoms and age, is important to create an optimal disease-specific intervention plan [21,26,27]. It is also crucial to identify and understand the unique characteristics of certain diseases; some diseases have a higher number of reported cases, but only a few patients have serious illnesses (eg, measles). Another electronic reporting system was used in Ireland to reduce the underreporting of notifiable diseases [28]. Underreporting has been associated with a number of factors, including clinicians’ lack of time and motivation and the use of complicated and inconsistent paper forms. The electronic system, which was consistent and user friendly, created an environment where clinicians could better engage in reporting and understand their notification obligations [21,25,29]. An interactive data visualization technique (eg, web dashboard) was also used to understand the unique characteristics of certain infectious diseases, thereby improving the accuracy and completeness of outbreak reporting [27]. Kamadjeu et al [30] developed an electronic dashboard for the poliovirus outbreak response in Somalia. The dashboard captured complete outbreak data and was able to improve the daily analysis and sharing of outbreak information between different stakeholders, such as outbreak response managers and immunization partners located in various areas.

The internet-based reporting system OSIRIS was introduced in the Netherlands to reduce delays in receiving outbreak data and improve the completeness of the data [20]. The system was able to reduce the delay from 10 days to 1 day and had a higher completeness of data with over 10% improvements compared with traditional paper-based approaches. Similarly, Angues et al [21] used a mobile-based data collection platform and real-time cloud-based storage technology to reduce delayed recognition and intervention. Fahnrich et al [23] also developed a mobile-based system that can efficiently and promptly control Ebola in West Africa.

**Table 1:** The descriptions of existing infectious disease surveillance systems found from our literature review.

Study	Infectious disease addressed	Description of main aims or features	Main outcomes reported	Fundamental requirements
Ward et al 2005 [20]	Meningitis, malaria, hepatitis A, B and C, etc	To improve timeliness and completeness of surveillance data on infectious diseases reported from regional to national level	The primary outcome measure was whether the internet-based reporting system improves timeliness and completeness in receiving the outbreak data in comparison with conventional paper-based forms. The internet-based system was able to reduce the delay and had a higher completeness of data.	Timeliness and completeness
Angues et al 2018 [21]	Nodding syndrome	To reduce delays in receiving outbreak data from rural regions in Uganda	The primary outcome measure was whether the real-time mobile-based system can improve the timeliness in reporting outbreak data. The system was able to accurately capture the data and reduce the delayed recognition and intervention. The system also created an environment where clinicians can better engage with the reporting and understand their notification obligations.	Accuracy, completeness, and timeliness
Severi et al 2014 [22]	Food poisoning, chickenpox, Q fever, etc	To improve sensitivity and timeliness of infectious disease data	An event-based surveillance system was developed. The reports from the system were timely and had complete information.	Timeliness and completeness
Fahnrich et al 2015 [23]	Ebola	To achieve efficient and timely control of communicable Ebola in West Africa	A standard digital Ebola outbreak management system was developed. The system can handle big data in combination with its mobile interface for bidirectional information exchange between different users.	Timeliness
Waarbeek et al 2011 [24]	Hepatitis, A, B, and C, etc	To design a real-time IDS^a^ system for cross-border collaboration	The collaborative disease protocols and risk assessments were embedded into the IDS system that provides real-time surveillance and geographical information.	Timeliness
Diwan et al 2015 [25]	Symptoms such as fever, cough, body ache, headache, and runny nose	To achieve efficient and timely collection of data in a resource-limited setting in rural India	The study demonstrated that a mobile-based system allowed to reduce the underreporting of notifiable diseases.	Accuracy and timeliness
Blanchon [26]	Acute diarrhea and influenza-like illness	To identify gaps in the French Sentinelles IDS system	The main finding is that it is important to maintain a high level of motivation and participation among physicians to improve the completeness and accuracy of outbreak data.	Accuracy and completeness
Yan et al 2013 [27]	Symptoms such as cough, sore throat, and fever	To design and pilot implementation of a syndromic surveillance system in rural China	A new web-based syndromic surveillance system was developed, which allows accurate and immediate reporting of syndromic data and automated detection of abnormal disease clusters using interactive visualization (ie, dashboard).	Accuracy, completeness, and timeliness
Brabazon et al 2015 [28]	Viral meningitis, viral encephalitis, bacterial meningitis, malaria, etc	To identify if there was significant underreporting of hospitalized notifiable infectious diseases in Ireland	A new computerized IDS system was developed, which reduced underreporting of outbreak data.	Accuracy and completeness
Karimuribo et al 2017 [29]	Any symptoms	To design a participatory disease surveillance system for community-based health reporters in East and Southern Africa	A mobile-based disease surveillance system was developed, which improved the engagement of people in their own communities in the detection of infectious human and animal disease threats.	Completeness
Kamadjeu et al 2017 [30]	Poliovirus	To design and implement an electronic dashboard for polio outbreak response management	The Somalia Polio Room Dashboard was developed; it integrates various outbreak and surveillance response data sources into rich graphical interfaces. The dashboard was able to reduce the need for daily analysis and sharing of outbreak information between different stakeholders.	Accuracy

^a^IDS: infectious disease surveillance.

### Phase 2: User Research and User Requirements

The results of the literature reviews allowed us to create a list of appropriate questions to be asked for subsequent face-to-face interviews. Some of the questions were designed to derive answers that could potentially help improve the ability to capture and report the outbreak data accurately, completely, and in a timely fashion. We conducted several interviews with 2 public health nurses and 1 clinical doctor from the PHU team to understand the overall workflow of how outbreak data were communicated between community facilities and the PHU team. The list of interview questions and their corresponding answers are presented in Table 2. Testimony from users and experts was used to help developers better understand user requirements.

**Table 2:** The list of interview questions and corresponding answers to understand the context of use.

Question ID number	Question themes	Interview questions	Answers (user requirements)	Sources
1	Data security and user access	Can anyone use the mobile app?	No; only registered clinicians should use the mobile app and the registration should be limited.	This was a common answer among all interviewees.
2	Data capturing	What are the required fields when entering outbreak data for a patient?	Patient name, date of birth, sex, vaccination details, symptoms and laboratory testing details, date of onset of symptoms, and date of last symptoms are required.	One of the public health nurses said some of the data such as laboratory testing details might not be available at the initial stage of the outbreak.
3	Data capturing (accuracy and completeness)	What are the most important fields (ie, mandatory fields) that need to be captured?	Patient names, case number, symptoms, symptoms onset date, sex, date of birth, disease type, and occupancy need to be at least captured.	This was a common answer among all interviewees.
4	Data update and review (timeliness)	How do you update and track the details of certain outbreak data?	Existing patient outbreak data must be reviewed and monitored to identify any changes in symptoms.	This was a common answer among all interviewees.
5	Data update and review (timeliness)	How often do you need to check the status of the outbreak or patients?	On the outbreak of infectious disease in a particular facility, the PHU^a^ nurses need to contact the facility every day to check the status of the outbreak and conditions of patients.	This was a common answer among all interviewees.
6	Data capturing (accuracy and completeness)	What are the common errors when entering outbreak data?	The symptoms of patients and symptoms onset date are often inconsistent. This is because there are multiple nursing staff at the facilities recording data every day, and there can be errors during handover.	This was a common answer among all interviewees.
7	Data report	What are the key fields that allow you to search and identify right patient?	Facility name, age, and patient name.	This was a common answer among all interviewees.
8	Data report	What are the fields of patients that you would like to see on the web dashboard? Would those fields be able to help you identify right information?	We felt that the most important field to see would be patient name, facility name, hospitalized (yes or no), deceased (yes or no), treatment or prophylaxis type, and pathogen (influenza or gastro). We thought these would be the most important to see first as long as full patient details can be provided from expanded page view if needed.	This was a common answer among all interviewees.
9	Data report	What are the most important reports that you would like to see when you log in to the web dashboard?	It is important to view the weekly total number of patients reported under each infectious disease for the past 6 months.	This was specifically requested by the clinical doctor.

^a^PHU: public health unit.

### Phase 3: Extraction of Function Requirements

**Figure 3:**
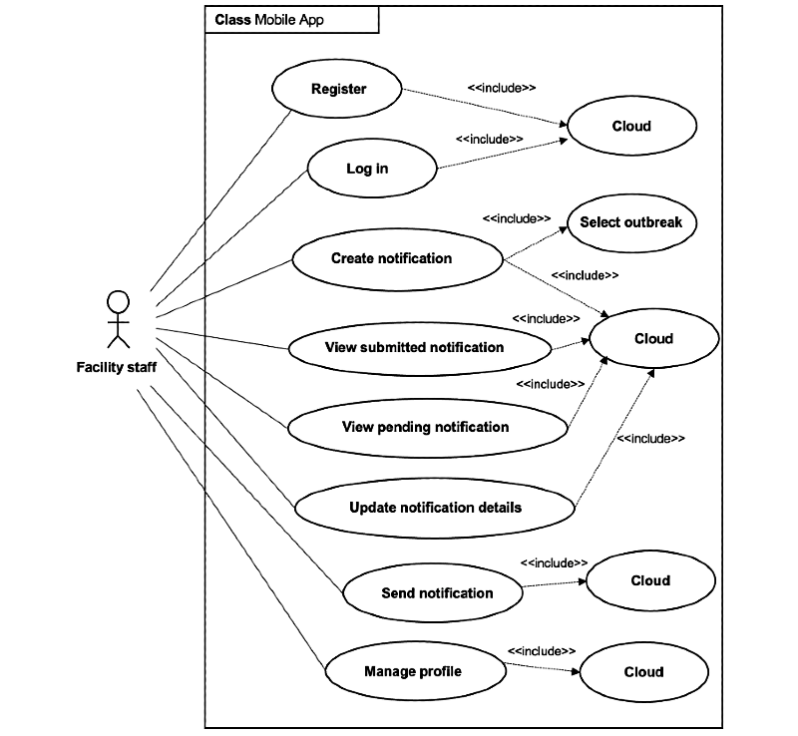
Use case diagram for the system (mobile app).

The results of the user interviews were then used to formulate functional requirements. A list of functional requirements is presented in Table 3. The use case diagram of our mobile app is shown in Figure 3. The diagram represents the main functionalities of the mobile app: data capturing and notification.

**Table 3:** The list of functional requirements formulated based on the outcomes of interviews.

Question ID number (consistent with Table 2)	Function type	Functional requirements
1	User verification for new registration	Send a request to the web server to verify the verification passcode entered by the user. If the passcode is validated, then the user can be registered; otherwise, display an error message.
2	Data capturing	Patient name, date of birth, sex, vaccination details, symptoms and laboratory testing details, date of onset of symptoms, and date of last symptoms. Validate the fields against the required fields. Show an error message if the user has not entered the mandatory field.
3	Data mandatory fields	Create mandatory fields on patient names, case number, symptoms, symptoms onset date, sex, date of birth, disease type, and occupancy.
4	Data update	Existing patient information can be easily retrieved, and users should be able to easily submit a new report based on previous outbreak data.
5	User alert for regular data submission	Create a push alert to inform users to regularly submit outbreak data for individual patient.
6	User error reducing functions	Most recently reported data can be prefilled, and the symptom onset date should not be editable once submitted.
7	User convenience functions	Create search bars for each field so that users can easily retrieve relevant information.
8	User report	Create an overview web page of selected infectious diseases and list all the patients in the table with the columns showing patient name, facility name, hospitalized (yes or no), deceased (yes or no), and treatment or prophylaxis type. Create an individual page to show all the details of the case reported when clicked by users.
9	User report	Create a table that displays the number of cases reported for each infectious disease during the outbreak; create landing pages that contain a 2-dimensional graph if there has been increase or decrease in the number of cases reported.

### Phase 4: Prototype Building and Iteration

On the basis of communication between clinicians and development teams, we developed an app and dashboard prototype. The app and dashboard were presented back to clinicians for further feedback, and we identified new user requirements that were not found in phase 1. A list of new requirements identified is shown in Table 4. We updated and improved the prototype system iteratively based on the feedback from 3 clinicians from the PHU team and 2 clinicians from the telehealth team over the period between January 2017 and December 2017. A description of the main functionalities of our prototype is presented in Table 5.

**Table 4:** The list of changes to functional requirements based on the exposure of prototypes to clinicians.

Question ID number	New functional requirements
8	Create a separate report designed for individual facility; summary of current active outbreaks within a facility. The data can also be visualized interactively, showing historical or specific details of an outbreak. Create a summary of current active outbreaks across the whole local health district. Specific outbreak details of certain facilities can also be visualized.
9	The data need to be better presented by highlighting important metrics such as the number of reported cases and patient details, including their symptoms and vaccinations. An automatic email notification to the PHU^a^ team is required when there are any new reported outbreaks.

^a^PHU: public health unit.

**Table 5:** The description of the main functionalities of our prototype.

Type	Main functionalities
Function 1: Data capturing	Outbreak details need to be entered, edited, and submitted using a mobile app or desktop PC (web interface) without geographical restrictions (within registered facilities managed by local PHU^a^). Clinical information collected includes patient name, date of birth, sex, vaccination details, symptoms and laboratory testing details, date of onset of symptoms, and date of last symptoms. Enforce correct and mandatory data entry (eg, facility care type and symptoms onset date).
Function 2: Outbreak notification	The data need to be sent and displayed in real time, highlighting important metrics such as the number of reported cases and patient details including their symptoms and vaccinations. An automatic email notification will be sent to the local PHU team.
Function 3: Data report for facilities	Summary of current active outbreaks within a facility. The data can also be visualized interactively, showing historical or specific details of an outbreak.
Function 4: Data report for PHU	Summary of current active outbreaks across the whole local health district. Specific outbreak details of certain facilities can be also visualized.
Function 5: Data storage	The data need to be processed and stored in a secure server.

^a^PHU: public health unit.

### System Overview

**Figure 4:**
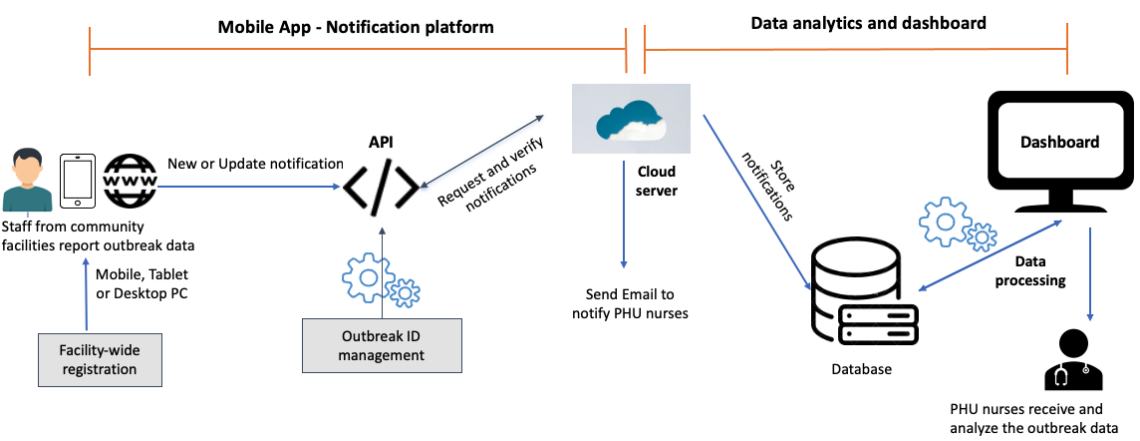
Overview of our proposed system. Data analysis and dashboard. API: application programming interface; PHU: public health unit.

Figure 4 shows an overview of our proposed system. Our system consists of a mobile app, a cloud server, and a dashboard. On the outbreak of an infectious disease in a facility, staff from the facility capture the patient data, including symptoms and vaccination details, using a mobile device or desktop PC (web interface) and send them to the cloud server. The data are then processed and displayed on the dashboard in real time. The cloud server automatically notifies PHU nurses by sending an email. The PHU nurses then use the web dashboard to receive and analyze the outbreak data.

#### Mobile App

**Figure 5:**
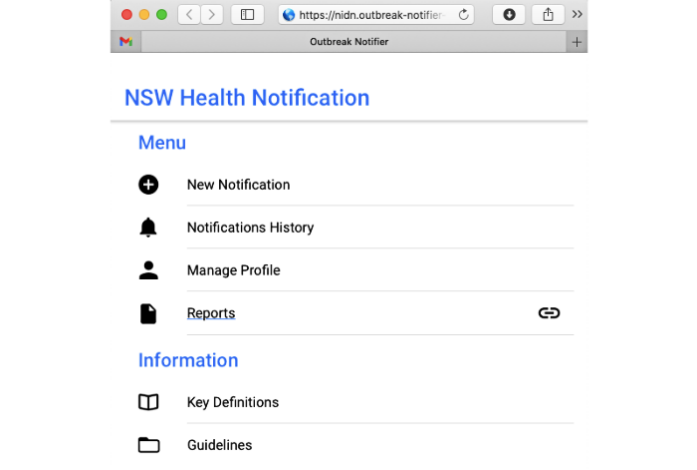
A web version of our mobile app with a consistent user interface. Web technologies such as HTML 5, Cascading Style Sheet 3, and JavaScript were used.

The mobile app was developed for both Android and iOS devices using a cross-platform app development framework. We specifically used a development platform called cross-compilation [31], which automatically transforms source code into platform-specific apps. We also developed a mobile web app so that it could be run in a standard web browser (Figure 5).

**Figure 6:**
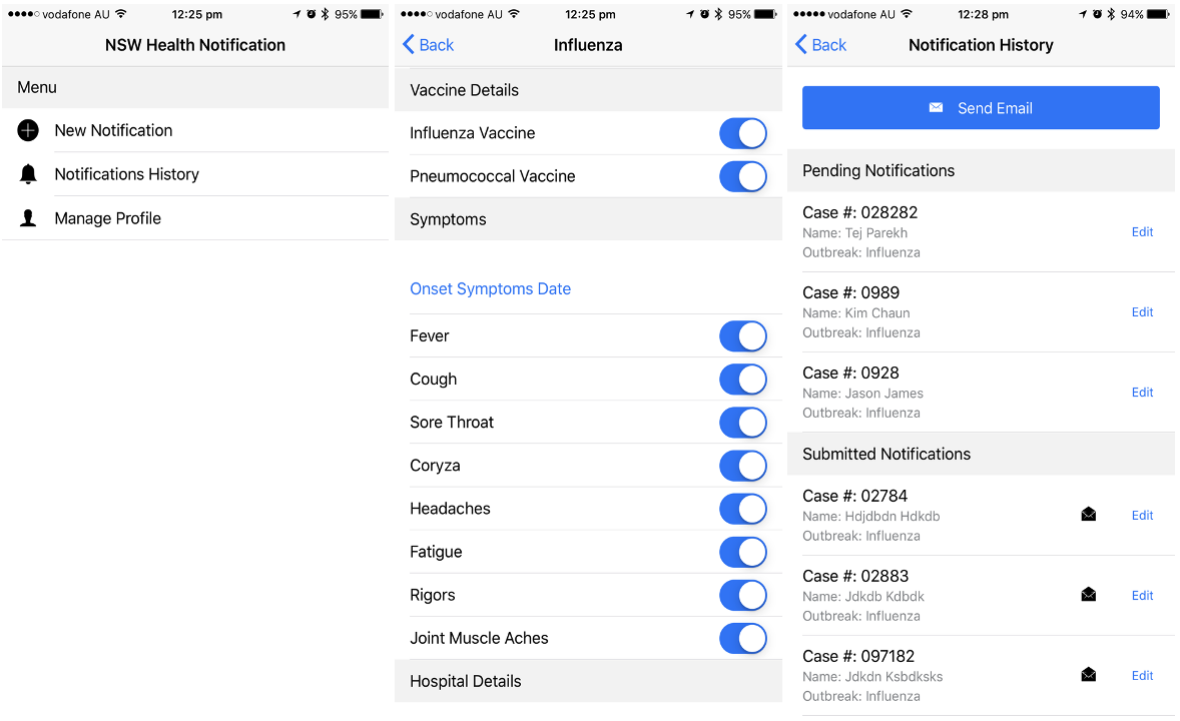
Example screenshots of our mobile app.

Users can create a new outbreak detail and add or edit the details of existing information, as shown in Figure 6 (function 1). During an outbreak of infectious diseases, it is important to monitor the symptoms of patients daily; therefore, our app allows us to review the history of previous patient details of outbreaks. Our app then sends them to a cloud server using an application programming interface gateway. To prevent unauthorized data submissions, the app is only accessible to users with a preregistered verification passcode, which is controlled by the PHU. All data are encrypted and sent using a JavaScript Object Notation (JSON) Web Token (JWT), which can only be validated at the cloud server (function 5). Automatic email notification is also sent to the PHU once a new outbreak is reported (function 2). The accessibility and user registration of the app are controlled by the cloud server. We used the local cloud server, and the data were encrypted using JWTs to enforce the security of communication.

#### Dashboard

We developed a dashboard using an open-source Hypertext Preprocessor (PHP) Laravel framework. It provides rich built-in features, such as authentication, authorization, and a database management system. The use of the Laravel framework allowed us to iteratively develop our system and efficiently refine it based on the feedback received from the PHU team.

**Figure 7:**
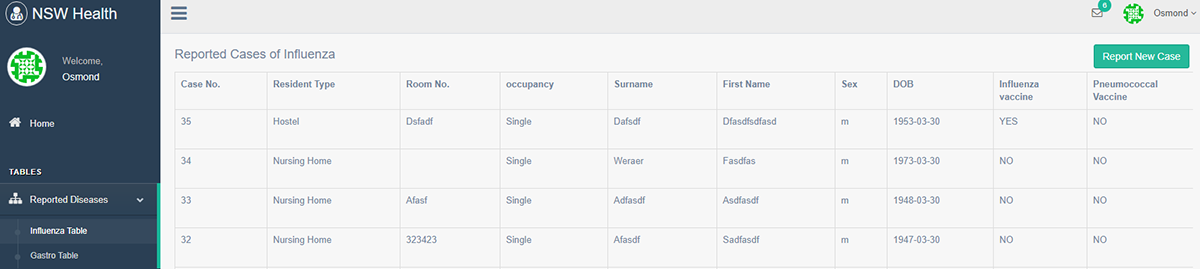
Details of the reported cases of infectious diseases. The Hypertext Preprocessor (PHP) Laravel framework is a popular and standard development framework that follows a model view controller architecture pattern.

**Figure 8:**
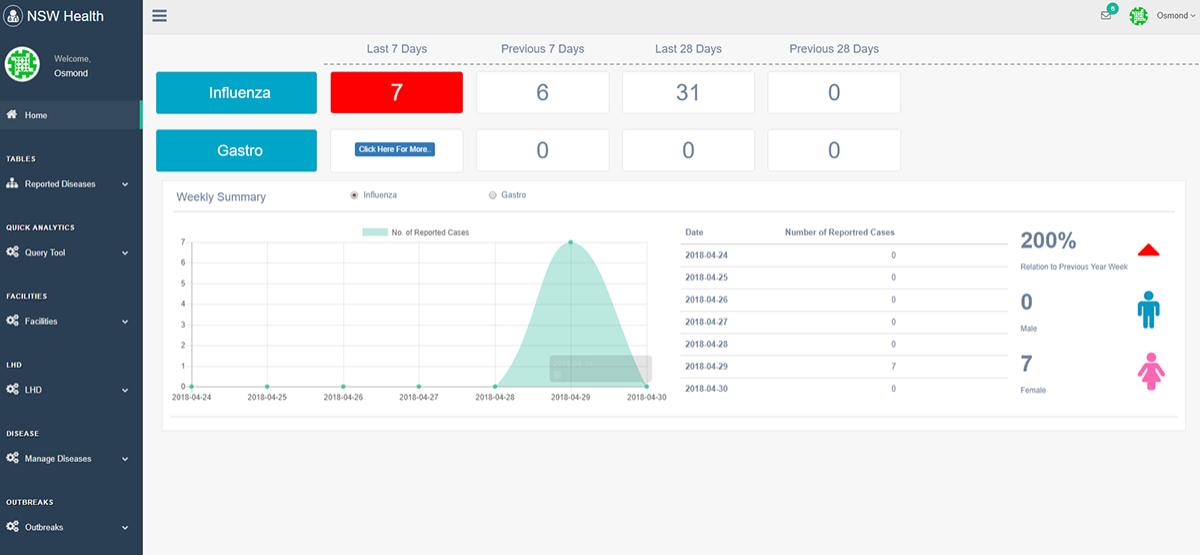
Daily, weekly, and monthly summary of current active outbreaks.

**Figure 9:**
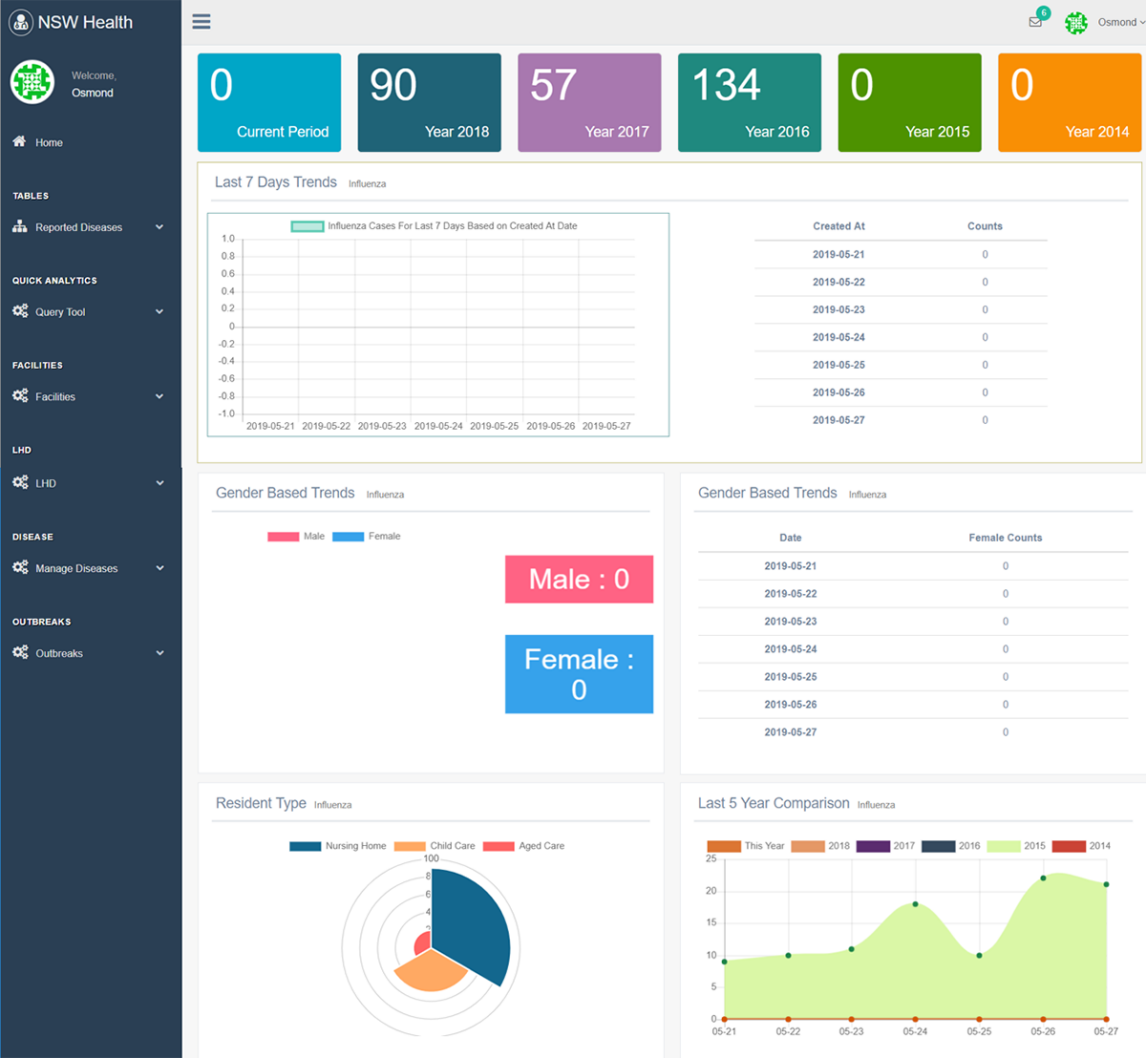
An historical summary of a specific outbreak.

The dashboard displays outbreak data sent via our mobile app in real time (function 2). Figure 7 displays a quick summary of the key information (eg, facility name, room number, patient details, and vaccination details). Figure 8 shows a summary of current active outbreaks, providing daily, weekly, and monthly summaries of reported cases (functions 3 and 4). To present data that are visually intuitive, key information is color coded and graphical charts are also used. More specific and historical data of an outbreak can also be retrieved. Figure 9 shows the trend of the selected outbreak over multiple years.

To make our system more applicable to wider domains (eg, different states or countries), data attributes such as different types of infectious diseases, symptoms, and registered community facilities can be added, modified, and deleted through the dashboard.

## Discussion

### Principal Findings

This study collected user requirements using a 2-pronged approach: a literature review and face-to-face interviews. We identified 3 fundamental requirements when designing an electronic IDS system through the literature review and developed a new IDS system using iterative face-to-face interviews. We also presented the development and implementation details of a new IDS system using mHealth technologies. Our study focused on the development of a generic mobile app or dashboard framework for capturing and reporting outbreak data, which is different from conventional existing works that are limited to addressing specific infectious diseases such as influenza [6,32-34] or patient cohorts [35].

With the portability of using a mobile device, outbreak data can be captured and sent from anywhere, and the use of the cross-platform framework further allows users to record the data with different types of devices (eg, mobile, tablet, and PC). The data are transferred using a client-server architecture and, therefore, can be analyzed in real time. Our dashboard is designed to provide a daily, weekly, monthly, and historical summary of outbreak information, highlighting important metrics such as the number of reported cases, male-to-female ratio, and number of reported specific symptoms. Specific information about certain outbreaks can also be visualized interactively, which can help understand the unique characteristics of emerging infectious diseases. Some of the key feedback we received from the PHU were as follows: (1) ability to enforce correct and mandatory data entry (eg, facility care type and symptom onset date) and (2) identification of key metrics that need to be visualized in the dashboard. The capability to customize data attributes is important for adaptively refining the system for general applications.

We suggest that the use of a mobile app and dashboard will simplify the overall data collection, reporting, and analysis processes, thereby improving the public health responses and providing accurate registration of outbreak information, which can potentially reduce associated morbidity and mortality [8]. This can also improve communication and engagement between clinicians and community facilities, providing more accessible information and education about infectious diseases.

We will evaluate the effectiveness of our proposed system by piloting the system at local community facilities, such as aged care and childcare centers. Preimplementation and postimplementation surveys will be conducted. Our system has a few limitations. Although our PHU team had extensive clinical experience, the system was developed and refined based on feedback from a single hospital; expanding across multiple hospitals will help generalize overall scopes.

### Conclusions

We suggest that accurate data reporting and collection, enabled by our system, are a major step forward in the early detection of a future outbreak of infectious diseases. With the availability of large-scale outbreak data, population-based analysis can be conducted to understand the overall characteristics of the outbreaks. This can potentially help prevent widespread infection and associated risks. Effective intervention plans can also be created based on the patterns identified and provided before the substantial avoidable spread of diseases has been incurred. Another key advantage of using large-scale data is the application of machine learning algorithms (eg, convolutional neural networks [36]) to predict infectious diseases. These predictive models can be derived and embedded in our notification system.

## Abbreviations

CSS: Cascading Style Sheet
IDS: infectious disease surveillance
JSON: JavaScript Object Notation
JWT: JSON Web Token
mHealth: mobile health
NBMLHD: Nepean Blue Mountains Local Health District
NSW: New South Wales
PHU: public health unit
